# Using physical activity levels to estimate energy requirements of female athletes

**DOI:** 10.20463/jenb.2019.0024

**Published:** 2019-12-31

**Authors:** Jonghoon Park

**Affiliations:** 1 Department of Physical Education, Korea University, Seoul Republic of Korea

**Keywords:** physical activity level, doubly labeled water method, estimated energy requirement, female athletes

## Abstract

**[Purpose]:**

The goal of this study was to review data on physical activity level (PAL), a crucial index for determining estimated energy requirement (EER), calculated as total energy expenditure (TEE, assessed with doubly labeled water [DLW]) divided by resting metabolic rate (RMR, PAL = TEE/RMR) in female athletes and to understand the methods of assessing athletes’ EERs in the field.

**[Methods]:**

For the PAL data review among female athletes, we conducted a PubMed search of the available literature related to the DLW method. DLW studies measuring TEE and RMR were included for the present review.

**[Results]:**

Briefly, the mean PAL was 1.71 for collegiate swimmers with moderate training, which was relatively low, but the mean PAL was 3.0 for elite swimmers during summer training camp. This shows that PAL can largely vary even within the same sport depending on the amount of training, and the differences in PAL were remarkable depending on the sport. Aside from the DLW method, there is currently no research tool related to athletes’ EERs that can be used in the field.

**[Conclusion]:**

Briefly, the mean PAL was 1.71 for collegiate swimmers with moderate training, which was relatively low, but the mean PAL was 3.0 for elite swimmers during summer training camp. This shows that PAL can largely vary even within the same sport depending on the amount of training, and the differences in PAL were remarkable depending on the sport. Aside from the DLW method, there is currently no research tool related to athletes’ EERs that can be used in the field.

## INTRODUCTION

In female endurance runners or gymnasts, chronic energy deficiency, when energy intake cannot meet the energy expenditure from high-intensity training, leads to amenorrhea and osteoporosis, creating the female athlete triad (FAT)^1,2^. These interrelated problems lead to not only decline in performance due to stress and chronic fatigue, but also sports injuries such as fatigue fractures^3-5^. To reduce the problems of the FAT, estimating daily calorie intake is essential for establishing detailed nutritional intake strategies^1,2^.

Currently, the doubly labeled water method (DLW) is the gold standard for measuring total energy expenditure (TEE) and calculating the estimated energy requirement (EER) in athletes^6^. A crucial index, EER is measured by the physical activity level (PAL) and the TEE divided by the resting metabolic rate (RMR). As individual RMR has a very narrow range of variance, the differences in the PAL determine the differences in the EER^6^. However, there are no reviews on PAL data obtained by the DLW method in female athletes or methods of calculating EER in athletes. Therefore, our study aims to review PAL data from DLW studies focusing on female athletes and to understand the methods of assessing athletes’ EERs in the field.

### Data review method

For the PAL data review among female athletes, we conducted a PubMed search of the available English literature related to the DLW method. DLW studies measuring TEE and resting metabolic rate (RMR) for calculating PAL were included for the present review.

### DLW method

The DLW method using stable isotopes of hydrogen and oxygen (2H and 18O) has been used worldwide in humans for about 60 years^7^. As the gold standard for TEE measurement, the DLW method is known as the most objective and accurate one^7-9^. When there is no weight change in the athlete during a week or two of the test period for obtaining DLW data, it is assumed that the TEE is identical to the daily EER^6^. The following briefly describes the DLW method used by our research team^6,10-14^. The DLW is produced by combining 10% ^18^O and 99.9% ^2^H, and the DLW composed of 1.2-1.8 g of ^18^O and 0.06 - 0.07 g of ^2^H is administered according to the recipient’s body weight. Urine samples are collected before DLW administration (baseline) and multiple times during the week. After the pretreatment process of vaporizing the isotopes through the equilibration method using catalysts such as platinum or zinc for hydrogen in the urine and carbon dioxide gas for ^18^O, the elimination rates of ^2^H and ^18^O are analyzed using an isotope-ratio mass spectrometer. Using the stable isotope ratio obtained from the urine samples collected at multiple time points after the administration of DLW, the elimination rates of ^2^H and ^18^O (k_h_ and k_o_) are calculated using a natural logarithm. The total body water (TBW) is calculated as the mean value of the dilution volume of ^2^H divided by 1.041, and the dilution volume of ^18^O is divided by 1.007. Carbon dioxide production is calculated by the equation rCO_2_ (mol/day) = 0.4554TBW (1.007k_o_ - 1.041k_h_), and TEE is calculated by the Weir equation TEE = 3.9(VCO_2_/food quotient (FQ))+1.1(VCO_2_)^15^ using carbon dioxide production and FQ estimated from the dietary intake investigation.

### PAL and EER predictions for non-athletes

To assess EER in the field, PAL is used as the most critical index. According to the Japanese standard of dietary intake, the EER is calculated as the RMR multiplied by the PAL (EER = RMR × PAL)^16^. In Korea, an EER equation following the US standard of dietary intake is used^6^. PAL acts as a major factor in EER data as the coefficient of physical activity (PA) in the EER equation, which varies depending on the differences in PAL^6^. For instance, if the PAL is around 1.6, a low active (1.11 and 1.12 for men and women, respectively) PA coefficient is applied, and if the PAL is 1.9-2.22, indicating high-intensity activities, a very active (1.48 and 1.45 for men and women, respectively) PA coefficient is applied.

EER for men and women aged 19 years and older (kcal/day):

Men: 662 - 9.53 × age (years) + PA [15.91 × weight (kg) + 539.6 × height (m)],

where PA = 1.0 (sedentary), 1.11 (low active), 1.25 (active), or 1.48 (very active).

Women: 354 - 6.91 × age (years) + PA [9.36 × weight (kg) + 726 × height (m)],

where PA = 1.0 (sedentary), 1.12 (low active), 1.27 (active), or 1.45 (very active).

In Japan, it was reported that Japanese adults have a low PAL distribution of around 1.6, and the normal range is around 1.75, although the data were not from a national representative sample^17^. In Korea, the majority of general adults have a low PAL distribution of around 1.46 in women and 1.55 in men, although the data were not from a national representative sample^18^.

### PAL data for female athletes

PAL data derived from the DLW method in Asian female athletes show that the mean PAL of collegiate tennis players was 1.97^19^, and the mean PAL of Japanese synchronized swimming athletes was 2.18^20^. PAL data of collegiate dinghy sailors during a training camp season show that the mean was 2.41^21^, and the mean of collegiate endurance runners was 2.68, both of which were relatively high^22^. PAL data in Western female athletes show that the mean PAL of basketball players was relatively high at 2.60^23^. The mean PAL of the US female endurance runners from a national team during its training period was 1.99, which was relatively low^24^, and the mean PAL of US female cross-country runners was 2.24^25^. The mean PAL of collegiate swimmers with moderate levels of training was relatively low at 1.71^26^, but the mean PAL of elite swimmers during summer training camp was 3.0^27^. Notably, the mean PAL of cross-country skiers was the highest among elite female athletes at 3.40^28^. This indicates that PAL among female athletes can vary largely, ranging from 1.71 to 3.40. Due to the number of cases with PAL far exceeding 2.20 in the sports event and training environment, there are limitations to applying the EER prediction method developed for the general public to athletes.

### EER prediction methods for athletes

Currently, there is no established method of calculating EER that considers the amount of training, except for estimating PAL using the DLW method. Although the DLW method is currently the most accurate for calculating EER, stable isotopes and the costs of the analysis are expensive, and it requires a high level of expertise to conduct the experiments, which makes it difficult to be applied in the field. Currently, to calculate EER in athletes, an equation developed by Japan Institute of Sports Sciences (JISS), which is to multiply 2.0 by RMR for athletes in ball games and to multiply 2.5 by RMR for endurance runners, is being used indiscriminately^29-31^. However, these calculations are restricted to one country, and these EER equations have the huge limitation of not considering the amount of training^32^. Currently, no countries have research methods related to EER that can be conveniently used for actively training athletes playing sports. For instance, if there is no weight change during one week of the test period, it can be assumed that TEE achieves balance with energy intake (EI), and therefore, EER calculation through EI measurement has been attempted^33^. However, large errors can occur during this EI measurement, and it is impossible to estimate the amounts of energy requirements during training, which fluctuate frequently.

Recently, Sagayama et al.^34^ measured the metabolic equivalents (MET) used in table tennis players during training and reported a total of 7 METs expended. Furthermore, the PAL of the table tennis players was a mean of 2.53 measured by the DLW method. Among the 7 METs, converting 6 METs (excluding 1 MET from resting) multiplied by 3 hours of training into PAL data yields approximately 0.75. Adding the PAL of 0.75 from training to a PAL of 1.75 from the average level of daily PAL yields a PAL of approximately 2.5, showing a similar value to the PAL of 2.53 calculated by the DLW method described above. Thus, Sagayama et al.^34^ suggested that it should be possible to develop an EER equation considering the amount of the intensity of training (METs) and estimated daily PAL. Recently, Yoshida et al.^22^ attempted to calculate EER by estimating the amount of training with the session-Rating of Perceived Exertion (RPE) method. To briefly describe the session-RPE method, it was developed by Foster et al.^35^ to quantify the amount of training, and it determines the RPE for each session and multiplies it by the training time. The session-RPE method is also used for endurance runners and swimmers^36,37^, and its validity and reproducibility have been proved^38^. Yoshida et al.^22^ attempted to estimate energy expenditure in endurance runners during training using the session-RPE based activity record, and they reported that the energy expenditure during training can be estimated with high accuracy. In addition, the energy expenditure from daily activities excluding training was estimated using a triaxial accelerometer. The accelerometer used by Yoshida et al.^22^ has the advantage of assessing the metabolism from daily physical activities with high accuracy using a unique algorithm that can precisely distinguish between locomotive and non-locomotive activity^39-42^. They reported that the TEE and PAL of endurance runners can be estimated with high accuracy (*r* > 0.8) using a combination of methods, using the session-RPE based activity record during training and the triaxial accelerometer to estimate daily energy expenditure^22^. Although the energy expenditure of endurance runners during training can be estimated using the heart rate method, this method requires expertise and the inconvenience of using a regression equation^9^. It is highly likely that the session-RPE based activity record can be very useful for not only assessing EER but also monitoring amounts of training and guiding endurance athletes. However, further studies on a larger number of subjects and sports are needed in order to apply the session-RPE based activity record in the field.

**Figure 1.**
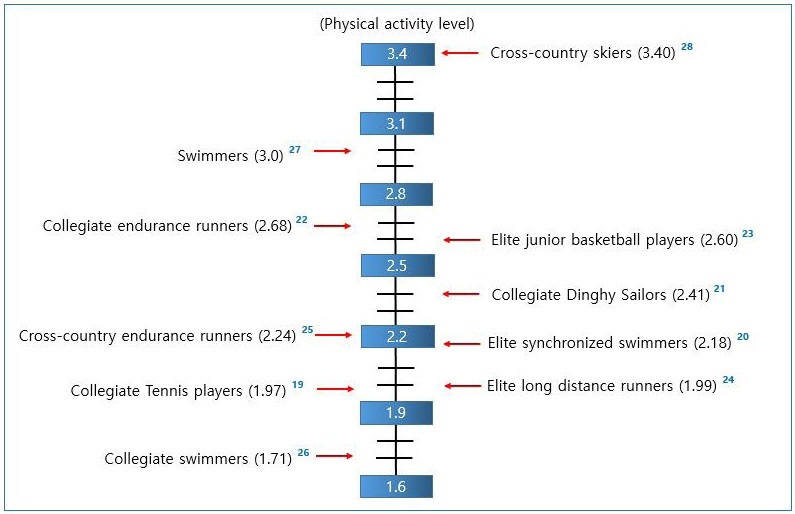
Physical activity levels (PALs) in female athletes. The data on PALs were derived from DLW studies identified in PubMed. Numbers in parentheses are PALs. Numbers in superscript are reference numbers.

## CONCLUSION

The present review describes research showing that PAL among female athletes was within a wide range of 1.71-3.40. Since the range of PAL in female athletes is very wide and it can exceed far over 2.20, depending on the event and training environment, there are limitations in applying the EER prediction developed for the general public to athletes. Further studies on PAL data from more diverse sports and environments during seasons or training camps obtained by the DLW method should be conducted, and the development of simplified measurement methods of predicting EER that can be easily used in the field is an urgent task.
